# Wide-Range Humidity–Temperature Hybrid Flexible Sensor Based on Strontium Titanate and Poly 3,4 Ethylenedioxythiophene Polystyrene Sulfonate for Wearable 3D-Printed Mask Applications

**DOI:** 10.3390/s23010401

**Published:** 2022-12-30

**Authors:** Adnan Ahmed, Afaque Manzoor Soomro, Darshan Kumar, Muhammad Waqas, Kashif Hussain Memon, Faheem Ahmed, Suresh Kumar, Hina Ashraf, Kyung Hyun Choi

**Affiliations:** 1 Department of Electrical Engineering, Sukkur IBA University, Sukkur 65200, Pakistan; 2 Department of Mechatronics Engineering, Jeju National University, Jeju-si 690756, Republic of Korea; 3 Department of Ocean Sciences, Jeju National University, Jeju-si 690756, Republic of Korea

**Keywords:** hybrid sensor, flexible device, wearable device, conductive polymer, 2D material

## Abstract

In this paper, we report a fast, linear wide-range hybrid flexible sensor based on a novel composite of strontium titanate (SrTiO_3_) and poly 3,4 ethylenedioxythiophene polystyrene sulfonate (PEDOT: PSS) as a sensing layer. Inter-digitate electrodes (IDEs) were printed for humidity monitoring (finger: 250 µm; spacing: 140 µm; length: 8 mm) whilst a meander-based pattern was printed for the temperature measurement (meander thickness: 180 µm; spacing: 400 µm) on each side of the PET substrate using silver ink. Moreover, active layers with different concentration ratios were coated on the electrodes using a spray coating technique. The as-developed sensor showed an excellent performance, with a humidity measurement range of (10–90% RH) and temperature measurement range of (25–90 °C) with a fast response (humidity: 5 s; temperature: 4.2 s) and recovery time (humidity: 8 s; temperature: 4.4 s). The reliability of the sensor during mechanical bending of up to 5.5 mm was validated with a reliable performance. The sensor was also used in real-world applications to measure human respiration. For this, a suggested sensor-based autonomous wireless node was included in a 3D-printed mask. The manufactured sensor was an excellent contender for wearable and environmental applications because of its exceptional performance, which allowed for the simultaneous measurement of both quantities by a single sensing device.

## 1. Introduction

Humidity and temperature are co-related environmental conditions with great importance in many industrial and domestic fields such as medical care [1], agriculture [2], food [3], information, and space [4]. The measurement and accurate control of such quantities have caused great concern for different biochemical, physical, natural, and artificial changings [5,6]. Regardless of the limit of detection, economical, accurate, and fast-responding humidity and temperature sensors and their hybrid sensors have been developed. They are based on resistive [7,8], capacitive [9,10], optical [11,12], acoustic [13,14], gravimetric [15], impeditive [16], and optical [17] changes with changes in temperature and humidity. The recent evolution of flexibility, wearability, and biocompatibility in humidity and temperature sensors has made these sensors applicable for human health-monitoring purposes such as body temperature [18], humidity, and inhaling and exhaling [19]. Furthermore, for the collection of non-invasive, real-time, and uninterrupted biological information, these sensors have been integrated with the multi-functionality of the Internet of Things (IoT) [20,21].

The characterization of hybrid sensors is highly dependent on the properties of the selected materials, which have been chosen by researchers to achieve better stability and reproducibility, a high sensitivity, and a rapid response and recovery time with certain economical fabrication methods [22,23]. The structure also plays a role in the fabrication; i.e., how the sensors are made to extract data in the required form. However, a few trade-offs have been made between the required ideal parameters such as the high power consumption, cost, and instability of highly sensitive optical humidity sensors compared with the quick response but expensive drive times of highly sensitive gravimetric sensors and the slow response times of highly sensitive capacitive humidity sensors [24]. For ideal results, novel materials with humidity- and temperature-sensing properties under various conditions have been studied such as Sn–NiFe2O4 [25], PEDOT:PSS composites [16,26], MOS_2_ and LiCl-doped TiO_2_ [27], WS_2_ [28], Si NCs [29], and two-dimensional materials GO and BP [26]. However, most of the researchers could not claim about the toxicity of the materials when exposed to human health-monitoring [30]. Therefore, biostable materials have been explored for such properties, e.g., biodegradable PLGA with a sensing range of (0–100% RH) and a response and recovery time of 3 s and 6 s, respectively [16]; collagen, a normally used protein proposed in a device with (50–90% RH) [31]; and a polysaccharide-built device with (30–80% RH) [32]. However, the limited applications, time, and cost have encouraged researchers to propose hybrid devices with wide ranges of simultaneously sensing humidity and temperature, along with a rapid response and recovery time.

In this paper, we present a hybrid flexible sensor containing a combination of a wide-range flexible humidity sensor and a wide-range temperature sensor for wearable human health-monitoring applications. As the active layer, a composite of strontium titanate (SrTiO_3_) and poly 3,4 ethylenedioxythiophene polystyrene sulfonate (PEDOT:PSS) was used. Inter-digitated-type sensor electrodes (IDEs) for humidity-sensing and meander-type electrodes for temperature-sensing were screen-printed on a flexible PET substrate. The hybrid sensor showed stable results in response to RH for the range (10–100% RH) and temperature for the range (25–90 ℃), with fast transient times of 3 s and 5 s and recovery times of 6 s and 9 s for humidity and temperature, respectively. The performance of the fabricated sensor was assessed in a custom-made electronically controlled chamber and a human-wearable mask was prepared to evaluate the practical human breathing application of the device. Furthermore, real-time and online patient breathing activity and body temperature-monitoring were analyzed with the advanced IoT-based functionality ThingSpeak API.

## 2. Methods and Experiments

For the fabrication of the hybrid sensor electrodes, a polyethylene terephthalate (PET) substrate with 200 µm thickness was purchased from Sigma Aldrich (St. Louis, MI, USA), silver conductive ink was imported from an ink bank supplier in the UK, and strontium titanate (SrTiO_3_) and poly 3,4 ethylenedioxythiophene polystyrene sulfonate (PEDOT:PSS) were purchased from the Shanghai Macklin Biochemical Co. Ltd. (Shanghai, China) and Shanghai D&B Biological Science and Technology Co. Ltd. (Shanghai, China), respectively. Furthermore, a silver epoxy paste (Sigma Aldrich) was used to make the connections to the sensor electrodes. Each of the solvent materials was dissolved separately into acetone and stirred for an hour at room temperature (32 ℃). By taking a specific ratio of 25 mg of SrTiO_3_ and 1 mL of PEDOT:PSS, a smooth solution was developed. Using a gravity spray gun, the resultant novel solution was sprayed as active layers of the hybrid sensor.

**Figure 1:**
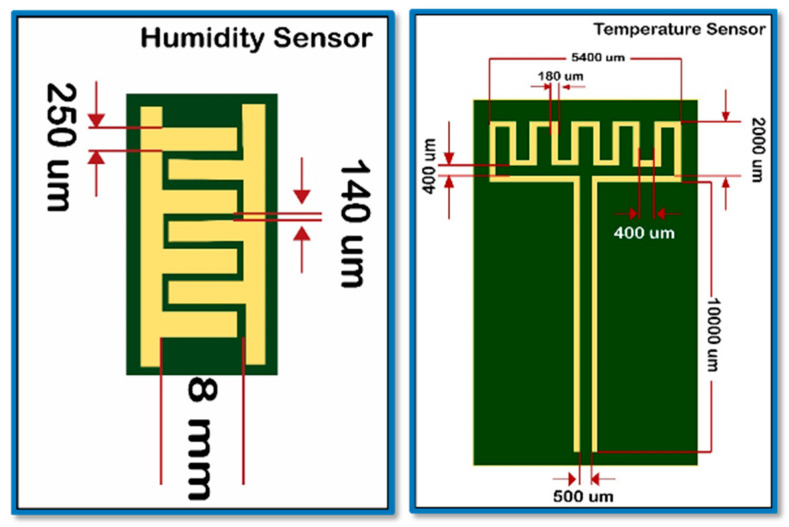
Design and dimensions of sensor electrodes for humidity sensor (**left**) and temperature sensor (**right**).

**Fabrication of the electrodes for the hybrid flexible sensor**. For the proposed hybrid sensor, inter-digitated electrodes were printed on one side of the flexible PET substrate for humidity-sensing and a meander-type pattern was printed on the other side for temperature-sensing. Each of the electrode-specific dimensions was designed on the 2D designing software Inkscape. The dimensions for each of the 7 fingers of the designed humidity sensor were 8 mm in length, with a 40 µm space between each electrode pair and 50 µm in thickness. The dimensions for the temperature sensor were 2000 µm in length, a 400 µm space, and 180 µm in thickness, as shown in Figure 1.

**Spray coating of the conductive slurry**. For the spray coating of the specified electrodes of the hybrid sensor, an active layer was created using SrTiO3 and poly 3,4 ethylenedioxythiophene polystyrene sulfonate (PEDOT:PSS) at a ratio of 25 mg and 1 mL, respectively. As an organic component with a high binding force, 1 mL of N, N-Dimethylformamide (DMF) was introduced into the prepared conductive slurry. As an active sensing layer, a properly mixed composite chemical layer was achieved using a hot-plate stirrer. The sensors coated with chemical layers were placed in a laboratory dry oven (DHA-9030A) at 70 °C for 2 h to ensure the optimum film treatment.

**Figure 2:**
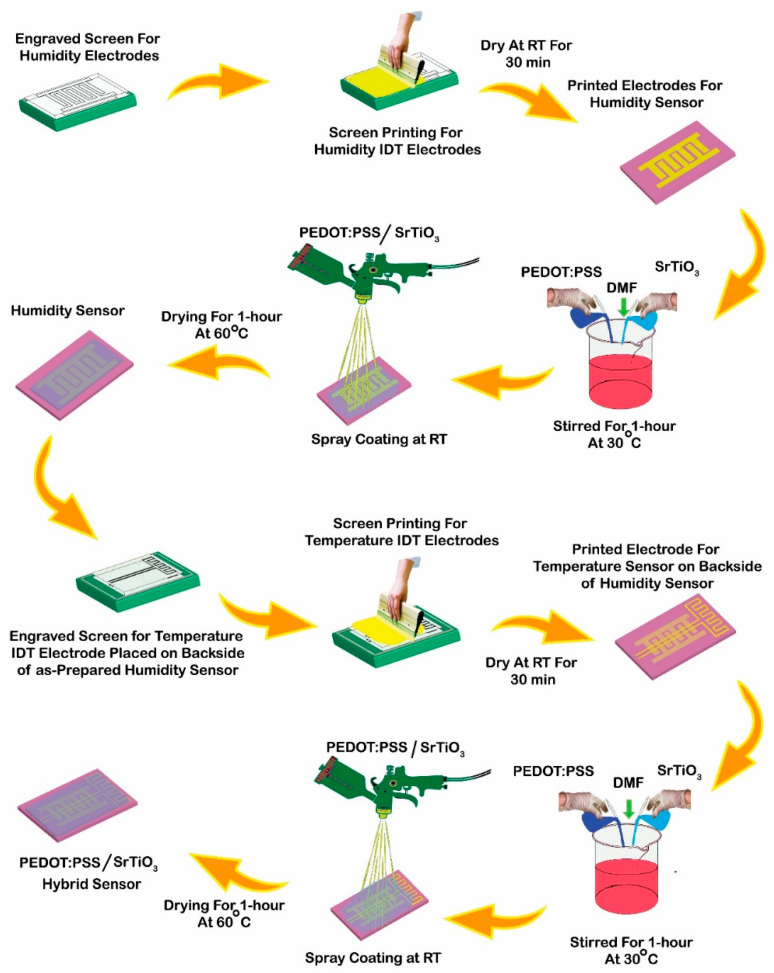
Fabrication process of the hybrid flexible sensor. Designed and engraved screens used for screen-printing of electrodes, printed electrodes coated with conductive slurry using spray coating method, and slurry prepared of SrTiO_3_ and PEDOT:PSS by adding DMF.

Figure 2 depicts the step-by-step fabrication procedure for the proposed hybrid flexible sensor. Later, the laser-cut engraved screens shown on the left for each humidity and temperature sensor were utilized for the screen-printing of the silver electrodes. After 0.5 h at room temperature, the sintering electrodes were confirmed. The dry IDT was then sprayed with a sensing layer. The coating compound polymer layer was composed of SrTiO3 and PEDOT:PSS materials, which were continuously stirred for an hour on a hot-plate stirrer at 30 °C. The sensor was then placed in the oven at 60 ºC for another hour to properly treat the coated film. The temperature sensor was made by repeating the processes. Furthermore, the integration of both the humidity and temperature sensors on a single PET substrate was demonstrated at the end. This was the proposed hybrid flexible sensor for this particular study.

**Figure 3:**
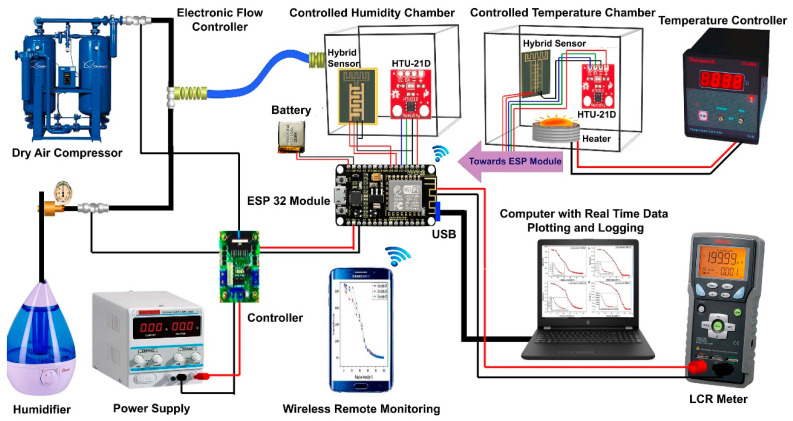
Characterization setup designed especially for measuring the sensor response.

**Sensor characterization**. The composition of the material used as the active layer was created using a LabRAM HR Raman spectrometer. A solid state laser of 514 nm was used as an excitation wavelength to acquire the Raman spectra. As shown in Figure 3, for the analysis of the electrical response of our hybrid flexible sensor, we developed a characterization setup. The custom-developed characterization setup for the measurement of the sensor response was designed to control humidity with a range of (10–100% RH) and temperature with a range of (25–90 °C). Using a Wi-Fi network with a USB-connected device, the sensor data were continuously monitored. As a PID feedback controller and reference sensor (HTU-21D), a commercial sensor was used. Other than data logging, an LCR meter was used to display the response of the real-time sensor to the computer against the change in impedance and capacitance of the sensor. Seeing the capacitive nature of the sensor, the meter was set to measure the values with five different frequencies (100 Hz, 120 Hz, 1 kHz, 10 Hz, and 100 Hz) with respect to time. The ESP32 module continuously transmitted the reference sensor data to the computer by using the USB communication device and the LCR meter.

With the exception of the dry gas compressor, which was manually operated, the in-house setup used loop-controlled inlets controlled by electronic mass flow controllers (MFCs). The dry air compressor was used to reduce the humidity to its lowest possible level of zero, and, by favoring it, the desired levels of humidity were attained. A smart air cooler was employed to lower the temperature. In contrast, a humidifier and an electronic heater were utilized, respectively, as humidity and temperature sensors to increase both quantities. The temperature of the chamber was maintained at 30 °C throughout the experiment because the ambient temperature would have had an impact on the observations. To receive the real-time and remote graphs of the impedance versus time results on a smartphone, an android application was designed with the help of an online source MIT app inventor.

## 3. Results and Discussion

The Raman spectra of the composite SrTiO_3_/PEDOT:PSS is shown in Figure S2. This could be divided into two regions: 250–450 cm^−1^ represented the first-order band and the second-order band was from 500 to 1150 cm^−1^. Herein, various modes of O-Sr-O (TiO_2_) and O-Sr-O (TiO_3_) were associated with those peaks. Moreover, a significant change was observed in the first-order band compared with the addition of PEDOT:PSS for the preparation of the composite material. However, the second-order showed the attributes for the prepared active layer. The relatively higher peak of 1095 cm^−1^ could have been related to the C-O out-of-plane bending vibration for PEDOT:PSS.

### 3.1. Working Mechanism

**Figure 4:**
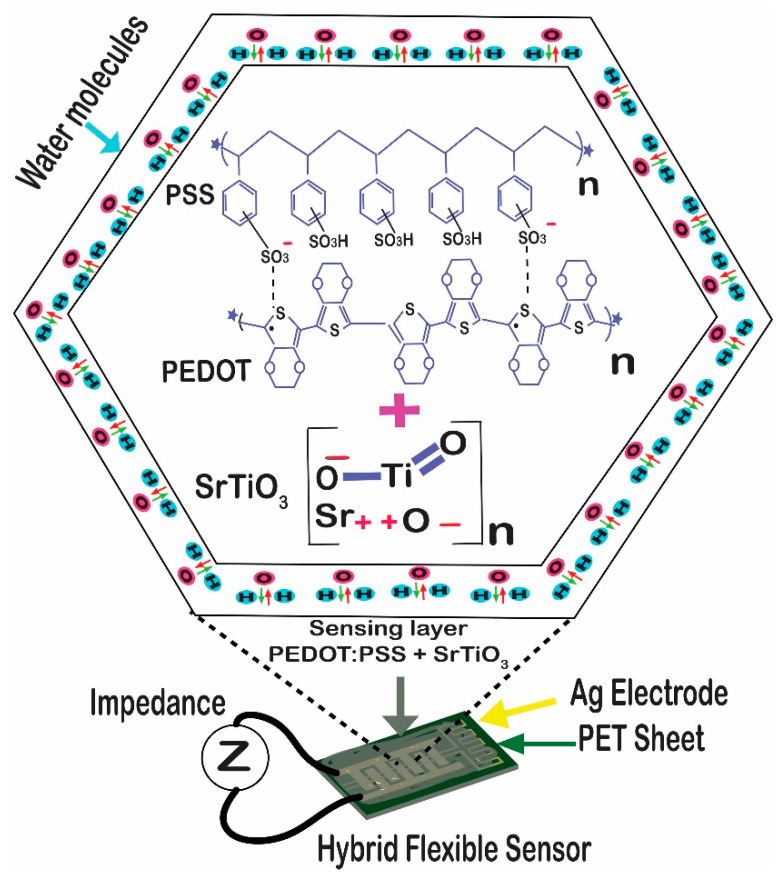
Working principle of the humidity sensor. For the temperature sensor, it was quite simple; an increase in temperature caused an increase in the impedance.

The chemical properties of the composite materials that were sprayed determined how our hybrid sensor functioned. Due to the hydrophilic behavior of the materials, changes in humidity and temperature tended to alter the impedance of the sensor. Both sensors displayed a capacitive and resistive shift in their response, but the impedance change had the main effect. The capture of the airborne water vapors of the detecting layer led to an increase in the conductivity of the sensor. The impedance was seen to decrease as a result. Additionally, in the case of temperature-sensing, the rise in impedance was compared with the rise in temperature. Figure 4 depicts the mechanism in detail.

The chemisorption and physisorption phenomena discussed here could each be used to describe the response of the proposed hybrid sensor. In the process of chemisorption, humidity rises and more water vapor is produced. The sensing layer traps such vapors, which enhances the ionic conductivity and triggers the protonic conduction, which initiates the current flow between electrodes. The use of physisorption of the sensing devices is a natural behavior [33,34].

### 3.2. Electrical Response

**Figure 5:**
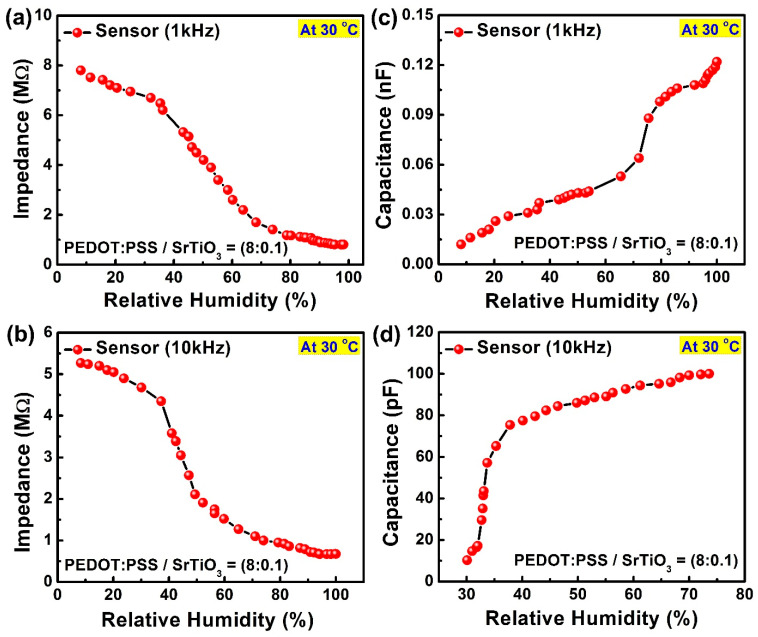
Electrical response of the sensor: (**a**) impedance versus relative humidity at 1 kHz; (**b**) impedance versus relative humidity at 10 kHz; (**c**) capacitance versus relative humidity at 1 kHz; (**d**) capacitance versus relative humidity at 10 kHz.

The reaction of the proposed hybrid sensor was based on the chemical characteristics of the sensing layers, including the structure of the sensor. The layers formed from the composite material drew heat and water vapor from the environment, comparatively enhancing the conductivity by lowering the impedance of the sensor. The decrease in the impedance of the sensor was inversely proportional to the rise in relative humidity and temperature. As conductive electrodes with a dielectric space in between, humidity-sensing IDEs have a structure that is both capacitive and resistive in nature. As a result, the impedance value was calculated in relation to changing humidity values, and temperature-resistive behavior was noted. The constructed sensors were cured for two hours at 70 °C prior to the electrical investigation. The electrical data were measured at a variety of frequencies, although the responses at 1 kHz and 10 kHz were more appropriate for this article. For the electrical characterization, the fabricated sensor was analyzed in a custom-developed electronically controlled chamber. The results showed that the change in the impedance was inversely proportional to the positive change in humidity and directly proportional to the change in temperature. The electrical response of the device is shown in Figure 5.

It was apparent from the analysis that the general behavior of the sensor was not the same throughout the range. There was a fast change in the impedance against the humidity; for large values of humidity, there was a small change in the impedance due to adsorption. The device showed an average change of 77.5 kΩ with each percent change in relative humidity. For the sensor at both frequencies (1 kHz and 10 kHz), the sensing pattern was significantly matched. The results shown in Figure 5a,b are given for the resulting impedance against the change in humidity at both frequencies of 1 kHz and 10 kHz, respectively. The experiment was performed under room conditions with a temperature of 30 °C. Capacitive–electrical results were observed for the changing capacitance against the change in humidity at 1 kHz and 10 kHz.

Figure 5c,d show the capacitive response against humidity, which was highly sensitive and linearly proportional at both frequencies. The sensor showed increasing response both at 1 kHz and 10 kHz in the range of nano- and pico-farads, respectively.

**Figure 6:**
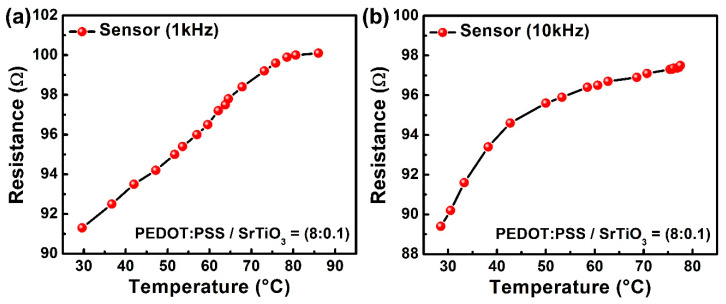
(**a**,**b**) Impedance versus temperature at 1 kHz and 10 kHz.

The outcomes shown in Figure 6a,b describe the experimental behavior for the temperature sensor at the same frequencies of 1 kHz and 10 kHz. For the temperature-sensitive side of the hybrid sensor, the electrical response was taken in the form of the impedance in contrast to the change in temperature. The composite layer was inherently highly sensitive to temperature; therefore, by increasing the temperature, the dryness increased, which ultimately increased the resistance in ohms. The resultant graphs illustrated the small variance in the impedance against the greater change in temperature. The device showed an excellent sensitivity of 0.3 ohms/°C.

**Figure 7:**
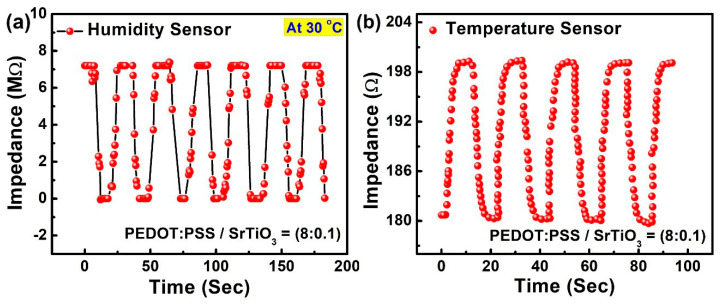
Cyclic response of the sensor: (**a**) relative humidity change from 10% RH to 90% RH; (**b**) temperature change from 30 °C to 80 °C.

**Figure 8:**
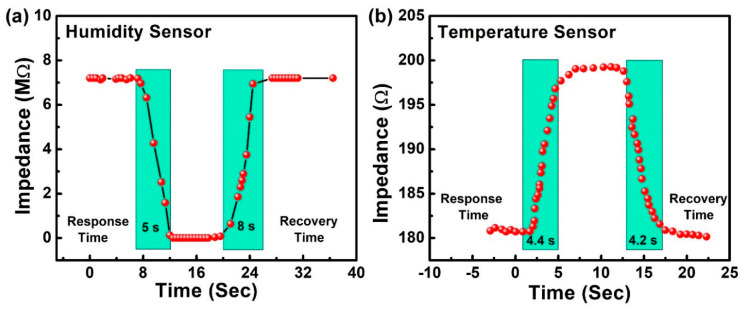
Response and recovery time of (**a**) humidity sensor and (**b**) temperature sensor.

The response and recovery times of a sensor are crucial, especially for any potential real-time applications. A repeated cycle of instantaneous humidification and dehumidification was applied to the e-controlled chamber in order to measure the transient response of the sensor with the response and recovery time. For that purpose, controlled quick-responding valves of a humidifier and a nitrogen gas cylinder were used [35,36,37]. Figure 7 shows the cyclic performance of the sensor with the transient response. The observed results of the sensor were in accordance with the real-time change. The response time of the sensor was the time taken by the sensor to respond to the change in relative humidity from 10% to 90%; the recovery time was the inverse of the process. The response and recovery time is shown in Figure 8. For this process, commercial sensors were used to determine the continuous changes in the RH and temperature. The cyclic process determined the outstanding results, with quick response times of 5 s and 4.2 s and recovery times of 5 s and 4.4 s for humidity and temperature, respectively. The results showed the emerging necessity of the hybrid sensor in diverse fields such as health-monitoring applications with such characteristics of fast response and recovery times.

### 3.3. Human Breath-Monitoring

**Figure 9:**
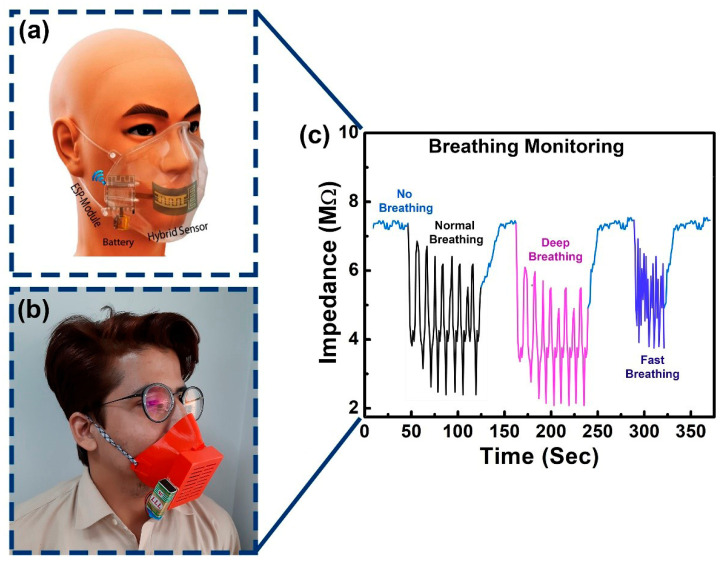
Implementation of the fabricated hybrid flexible sensor for a wearable human health-monitoring application. (**a**) shows the proposed prototype, (**b**) shows the subject wearing the as-developed gear, (**c**) shows the response of breathing.

The sensor, as created, was eventually utilized to track the breathing activity of a human. A wearable mask was 3D-manufactured for this purpose, as illustrated in Figure S2. The custom-built arrangement could power a readout circuit with a portable battery, and functioned as a wireless node. The data were delivered to a cell phone in real-time, and a further examination in accordance with medical norms was possible. The sensor was placed inside the mask to monitor the respiratory response, as shown in Figure 9. The mask was used by a volunteer subject in normal room conditions. The conformal design of the ready-to-use apparatus caused a significant level of comfort, with no effect on the natural response of the user. The response was categorized into four different styles of breathing: routine breathing; deep breathing; fast breathing; and a deliberate delay between two respective breathing methods. The user was already guided regarding the apnea phase to also test the fabricated sensor across its possible dynamic applications. The outcomes showed a significant variation in impedance due to deep breathing as, in this condition, more water vapors were exhaled out and detected by the sensor. Figure 9 shows the corresponding application of the sensor for the respirational response of a human being. Here, the results of the hybrid sensor were observed at 1 kHz. Each of the possible four breathing styles were highly distinguishable such as apnea (~6.65 M Ω), normal breathing (~6 M Ω), deep breathing (~5.2 M Ω), and fast breathing in a range from 3 Ω to 6 Ω. The proposed device was a strong and sustainable candidate to detect human respiration with significant efficiency and accuracy.

**Figure 10:**
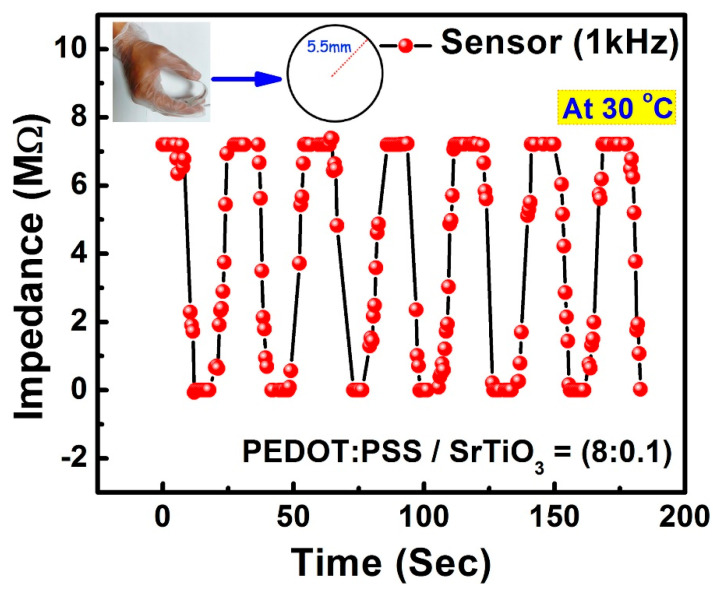
Response of the sensor to humidity-sensing under continuous mechanical bending with a radius of 5.5 mm.

The human body, which is made up of joints wrapped in stretchy skin, necessitates flexible devices that are not impacted by the flexibility or stretchability of the skin. As a result, preferable gadgets in human health-monitoring systems are those whose results are unaffected by bending. Herein, the silver conductive electrodes for both humidity and temperature were fabricated on a flexible PET substrate. The performance of the sensor was stable, linear, sensitive, and reliable with a bending radium of 5.5 mm, which showed the mechanically analyzed flexibility of the sensor. This mechanical evaluation supported the application of the sensor in a wearable invasive and non-invasive medical aspect. The mechanical bending process and unaffected observations are shown in Figure 10. The response of the sensor under continuous bending was highly consistent with that of the normal physical state.

Altogether, a flexible biocompatible hybrid sensor with a fast response and recovery time that was highly sensitive to large ranges in humidity and temperature is reported here. The results defend its performance in many fields of work, particularly related to health-monitoring applications.

## 4. Conclusions

In this paper, we designed, built, and tested a hybrid flexible sensor with high linearity and stability, and a wide sensing range for humidity and temperature. The electrodes of the hybrid sensor were created using a screen-printing process. As the sensing layers for the hybrid sensor, thin films of a new combination of SrTiO3 and PEDOT:PSS were used with a spray coating on the electrodes. The electrical, chemical, and mechanical characteristics of the sensor were examined to evaluate the properties. The response and recovery times of the sensor in respect to humidity were 5 s and 8 s, respectively; these were 4.2 s and 4.4 s in the case of the temperature sensor. The fabricated sensor was mechanically stable with a consistent response to bending at a radius of 5.5 mm, showing an excellent sensitivity with 77.5 kΩ/% RH at a wide range of 10–90% RH. Moreover, the sensitivity of 0.3 ohms/°C was recorded from 30–80 °C. For the practical application of the as-developed sensor, a mask was 3D-printed, which carried all the necessary components to be a wireless breath-monitoring apparatus in addition to the sensor. The sensor could easily distinguish between different breathing styles, enabling it to be a strong commercial candidate for wearable health-monitoring applications.

## Data Availability

Not applicable.

## Supplementary Materials

The following are available online at https://www.mdpi.com/article/10.3390/s23010401/s1, Figure S1: 3D printed mask, Figure S2: Raman scattering spectra, Figure S3: flexibility test, Figure S4: immunity test of humidity sensor, Figure S5: immunity test of temperature sensor
