# Supplementary material for: Wide-Range Humidity–Temperature Hybrid Flexible Sensor Based on Strontium Titanate and Poly 3,4 Ethylenedioxythiophene Polystyrene Sulfonate for Wearable 3D-Printed Mask Applications
Source: Sensors (Basel). 2022 Dec 30;23(1):401. doi: 10.3390/s23010401 (PMC9823765; doi:10.3390/s23010401)
Supplement: Supplementary file 1 [file sensors-23-00401-s001.zip › sensors-1855693-supplementary.pdf]

Supporting Information for

Wide Range Humidity-Temperature Hybrid Flexible Sensor Based on Strontium Titanate and Poly 3,4 Ethylenedioxythiophene Poly Styrene Sulfonate for Wearable 3D Printed Mask Application

Adnan Ahmed[a,+], Afaque Manzoor Soomro[c,*], Darshan Kumar[a,+], Muhammad Waqas[a,*], Kashif Hussain[a], Faheem Ahmed[c], Suresh Kumar[a], Hina Ashraf[b] , Kyung Hyun Choi[c]

[a]Department of Electrical Engineering, Sukkur IBA University, Pakistan,
[b]Department of Ocean Sciences, Jeju National University, Republic of Korea,
[c]Department of Mechatronics Engineering, Jeju National University, Republic of Korea,
**Correspondence and request for materials should be addressed to Muhammad Waqas & Afaque Manzoor Soomro (Email addresses: mwaqas@iba-suk.edu.pk; afaquemanzoor@gmail.com_) [+]Both Authors contributed equally*

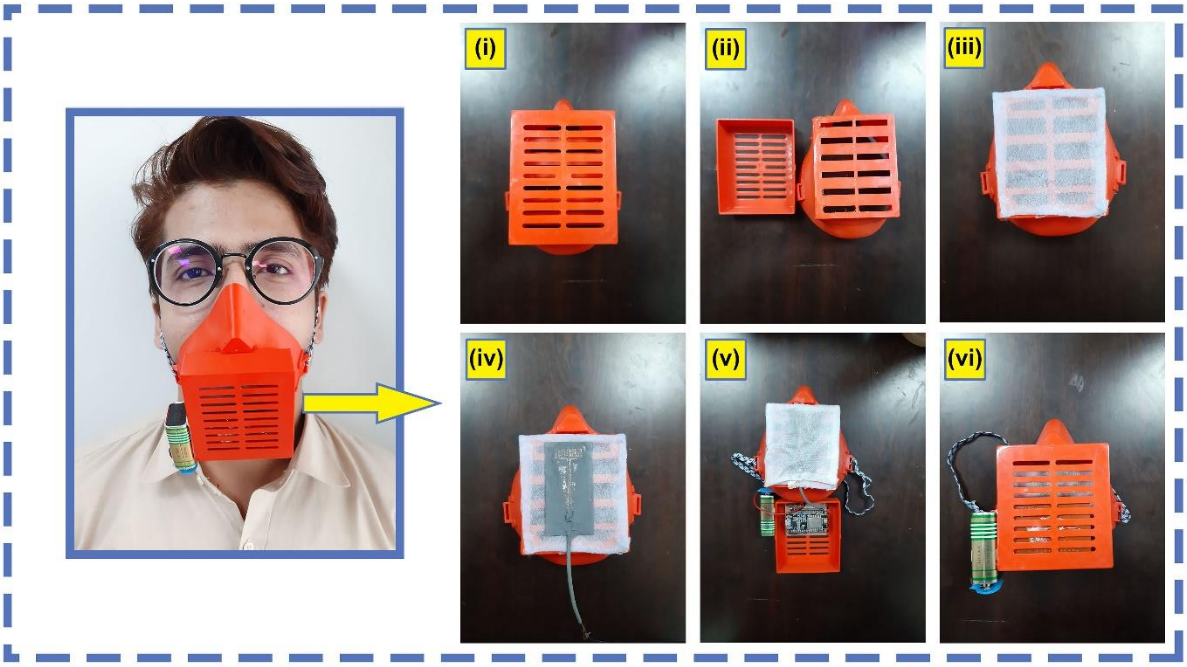

Figure S1. Custom made 3D printed mask, with capability of holding the fabricated sensors and its readout circuit along with a portable battery; real images of the prepared sample i) printed mask, ii) two individual parts (the left one is used to hold the sensor), iii) placement of a commercial mask used for COVID-19, iv) placement of sensor, v) placement of wireless node, vi) placement of portable battery.

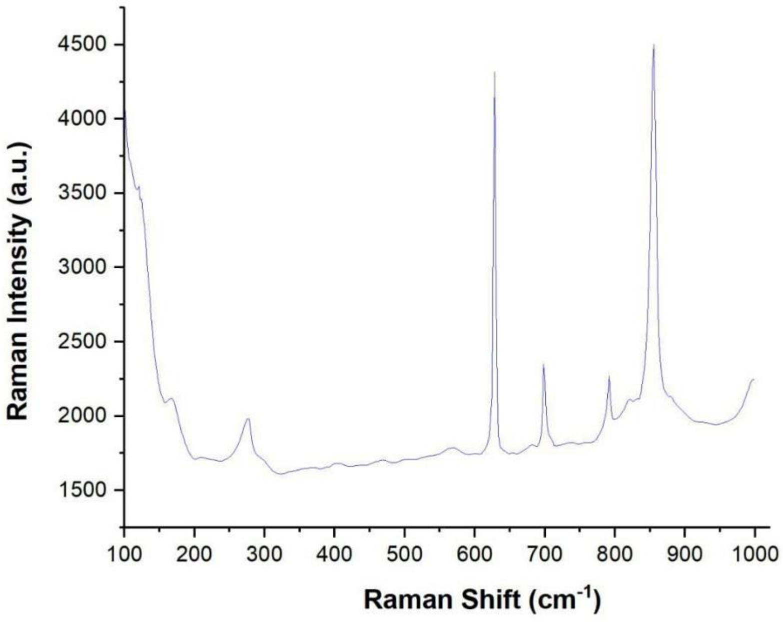

Figure S2 Raman scattering spectra

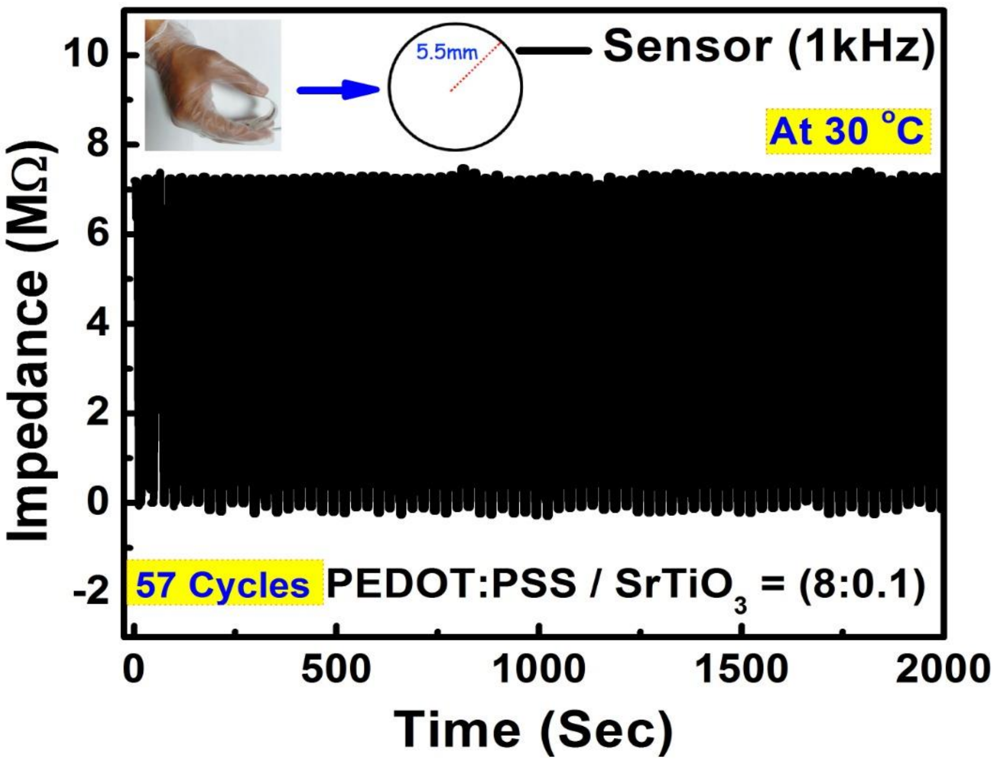

Figure S3 Flexibility test for 57 cycles.

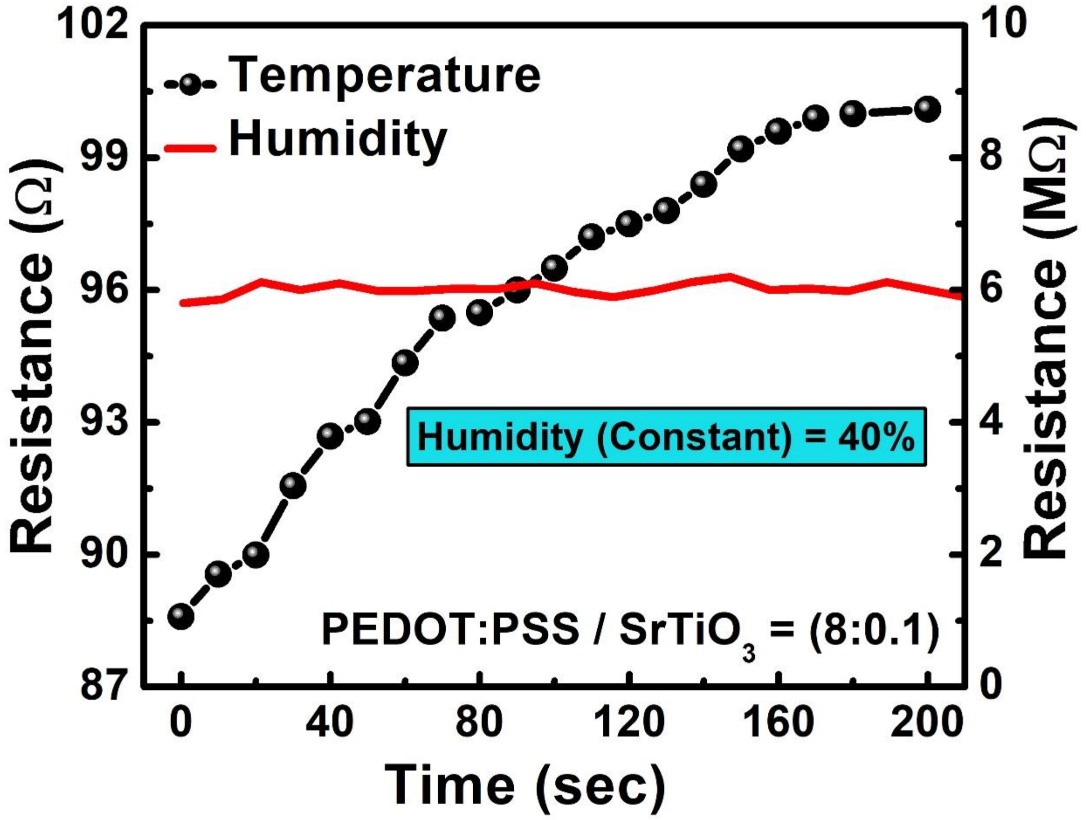

Figure S4 immunity test of humidity sensor against temperature changes.

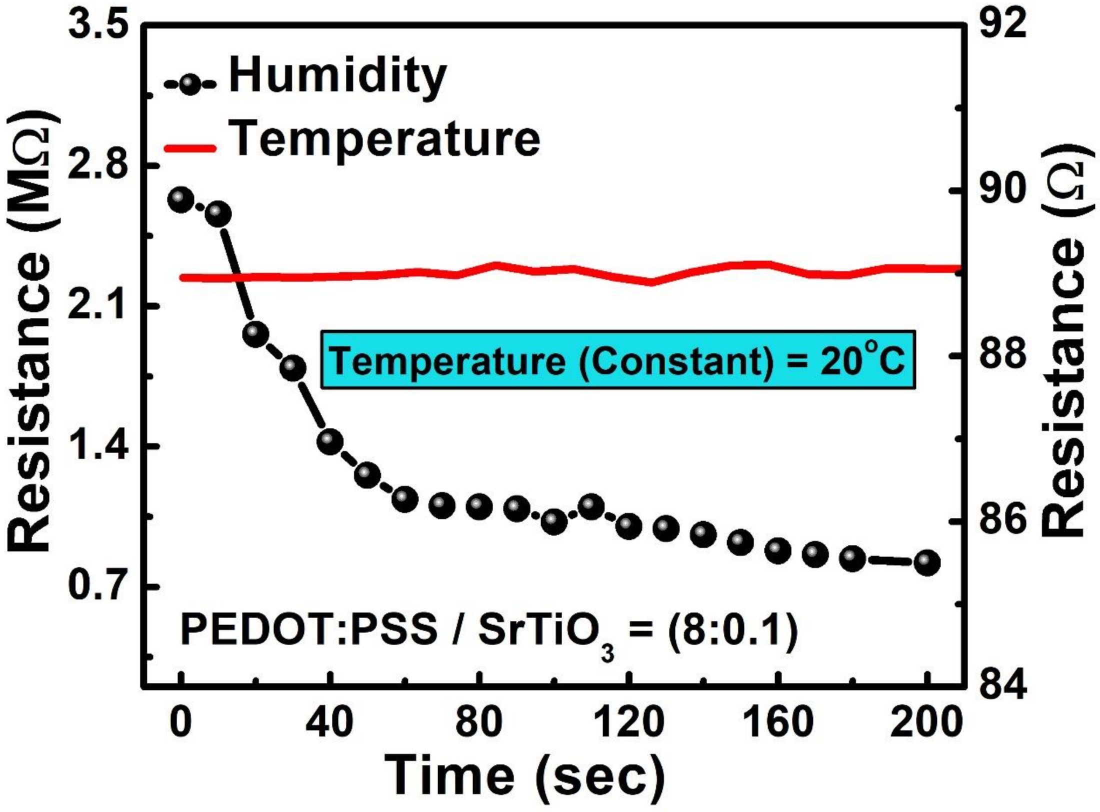

Figure S5 immunity test of temperature sensor against humidity changes.
